# Corticotropin-releasing hormone is significantly upregulated in the mouse paraventricular nucleus following a single oral dose of cinnamtannin A2 as an (−)-epicatechin tetramer

**DOI:** 10.3164/jcbn.19-19

**Published:** 2019-06-07

**Authors:** Yasuyuki Fujii, Kenta Suzuki, Takahiro Adachi, Shu Taira, Naomi Osakabe

**Affiliations:** 1 Department of Bioscience and Engineering, Shibaura Institute of Technology, 307 Fukasaku, Minuma-ku, Saitama 337-8570, Japan; 2 Department of Immunology, Medical Research Institute, Tokyo Medical and Dental University, Tokyo 113-8510, Japan; 3 Fukushima University, Faculty of Food and Agricultural Sciences, 1 Kanayagawa, Fukushima 960-1248, Japan

**Keywords:** cinnamtannin A2, stress, hypothalamic-pituitary-adrenal axis, corticotropin-releasing hormone, c-fos

## Abstract

Cinnamtannin A2, an (−)-epicatechin tetramer, was reported to have potent physiological activity. Cinnamtannin A2 is rarely absorbed from the gastrointestinal tract into the blood and the mechanisms of its beneficial activities are unknown. Cinnamtannin A2 reported to increase sympathetic nervous activity, which was induced by various stressors. In present study, we examined the stress response in the mouse paraventricular nucleus following a single oral dose of cinnamtannin A2 by monitoring mRNA expression of corticotropin-releasing hormone (CRH) and c-fos using *in situ* hybridization. Corticotropin-releasing hormone mRNA showed a tendency to increase at 15 min and significantly increased at 60 min following a single oral administration of 100 µg/kg cinnamtannin A2. After a single dose of 10 µg/kg cinnamtannin A2, there was significant upregulation of CRH mRNA at 60 min. These results suggested that cinnamtannin A2 was recognized as a stressor in central nervous system and this may lead to its beneficial effects on circulation and metabolism.

## Introduction

**Fig. 1:**
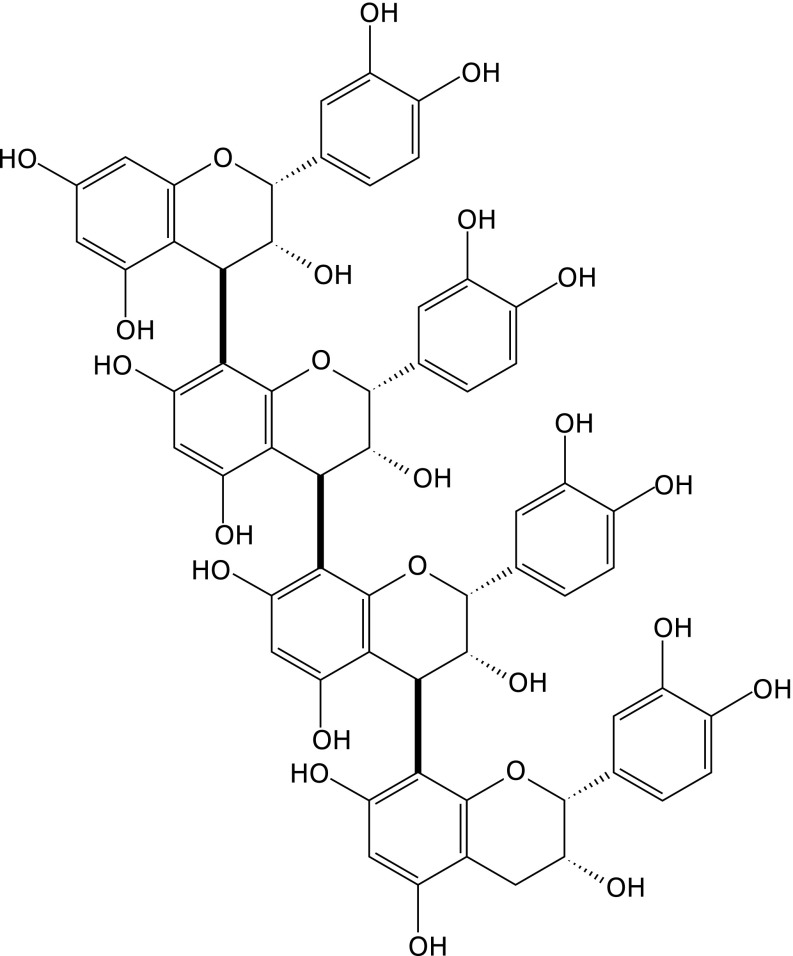
Chemical structure of cinnamtannin A2.

Cinnamtannin A2 (A2) is a B type procyanidin that is an (−)-epicatechin tetramer, linked by C4–C8 bonds (Fig. 1). A2 is present as an astringent substance in chocolate, red wine, immature apples, pine bark, and some other foods with (−)-epicatechin.^(1–3)^ Foods rich in procyanidins have shown significant potential in managing cardiovascular health.^(4–6)^ Recent reports also suggested that ingestion of procyanidin-rich foods improved cognitive function in elderly volunteers.^(7,8)^ In particular, it was reported that mood, cognitive performance, and working memory were improved soon after ingestion of procyanidin-rich foods, along with an increase in cerebral blood flow.^(9–12)^ These previous reports suggested that ingestion of procyanidins directly or indirectly affected the central nervous system. However, these mechanism of action are still unknown.

A recent comprehensive review on polyphenols and human health indicated that the mechanism underlying these beneficial effects of procyanidin-rich foods has not been fully elucidated, due to their poor bioavailability.^(13–15)^ The B-type procyanidins are rarely absorbed from the gut into the blood.^(16–18)^ In spite of this poor bioavailability, A2 had potent activity among the B-type procyanidins. For instance, A2 showed strong activity on glucose transporter 4 translocation from internal membrane pools to the plasma membrane in skeletal muscle.^(19,20)^ A single oral administration of 1 µg/kg A2 significantly elevated uncoupling protein-1 mRNA in brown adipose tissue; while, procyanidin B2 (dimer) or procyanidin C1 (trimer) showed similar activity at a high dose of 1,000 µg/kg.^(21)^ In addition, a high dose of A2 (100 µg/kg) did not lead to UCP-1 upregulation alone, it only did when combined with an α2 adrenaline blocker. Since the α2 adrenergic receptor, an inhibitory receptor, is present mainly in the central nervous system, those results suggested that a single oral dose of A2 affected the central nervous system and consequently elevated sympathetic nerve activity.

Generally, when an organism is exposed to stress, the body has an adaptive response caused by activation of the sympathetic-adrenal-medullary (SAM) axis along with activation of the sympathetic nerve and the hypothalamic-pituitary-adrenal (HPA) axis.^(22)^ The enhancement of sympathetic nerve activity seen after administration of A2 was thought to be due to activation of the SAM axis. It was also suggested that the HPA axis was activated by A2 treatment. In the present study, we examined that A2 causes a stress response in the HPA axis by monitoring changes in mRNA expression of c-fos, a neural activity marker, and corticotropin-releasing hormone (CRH), a stress hormone, in the paraventricular nucleus (PVN) in the hypothalamus using the method of *in situ* hybridization.

## Methods and Materials

### Animals

The study was approved by the Animal Care and Use Committee of the Shibaura Institute of Technology (Permit Number: 27-2956). All mice received humane care under the guidelines of this institution. The C57Bl/6 (12 weeks old) mice were purchased from Charles River Laboratories, Japan, Inc. (Tokyo, Japan). All animals were housed in a room maintained under standard conditions of light (12/12 h light/dark cycles), temperature (23–25°C), and humidity (50 ± 5%), with *ad libitum* water and basal diet (MF^®^; Oriental Yeast Co., Ltd., Tokyo, Japan).

### Materials

A2 was obtained from Phytolab GmbH & Co., KG (Vestenbergsgreuth, Germany). The other chemicals were purchased from Wako Pure Chemicals (Tokyo, Japan).

### Experimental procedures

Mice were divided into 10 treatment groups, as follows: before (no treatment, *n* = 8), vehicle (3% ethanol, 10% Tween80, in phosphate buffered saline [PBS]), 10 µg/kg A2 (*n* = 4–6), and 100 µg/kg A2 (*n* = 5–6). The A2 was suspended in vehicle. At 15, 60, and 120 min after administration of vehicle or A2, mice were anesthetized with pentobarbital (50 mg/kg body weight, administered i.p.; Tokyo Chemical Industry, Tokyo, Japan). We used a gastric tube with round edges, an infusion rate of 1.0 ml/min, and maintained the temperature of administration solution at 37°C to avoid any stress induced by this procedure. Then, mice were perfused through the left ventricle with 25 ml PBS and then with 25 ml 4% paraformaldehyde (PFA) in PBS for fixation. Brains were immediately removed and placed in a post-fix solution of 4% PFA at room temperature.

### *In situ* hybridization

Whole brains were dehydrated with ethanol, immersed in xylene, and subsequently embedded in paraffin. Paraffin coronal sections (8-µm thick) of the PVN were prepared from −0.3 mm bregma with a microtome (Erma, Tokyo, Japan). *In situ* hybridization was carried out to evaluate c-fos and CRH mRNA expression with the RNAscope^®^ 2.5HD Duplex Assay (Advanced Cell Diagnostics, CA) according to our previous report.^(23)^ Sections were observed with an Olympus CX41 light microscope (Olympus Co., Tokyo, Japan), equipped with a digital camera, D5300 (Nikon Co., Tokyo, Japan). We quantified the *in situ* hybridization results by counting the cells labeled with probes that targeted CRH and c-fos mRNA using NIH Image J software (http://rsb.info.nih.gov/ij/index.html, 11 June 2018) according to our previous report.^(23)^

### Statistical analyses

All data are expressed as the means ± SEM. Statistical analyses were performed with two-way ANOVA, followed by the post hoc Dunnett’s test. *P*<0.05 was considered significant when comparing the vehicle and A2 treatment groups.

## Results

**Fig. 2:**
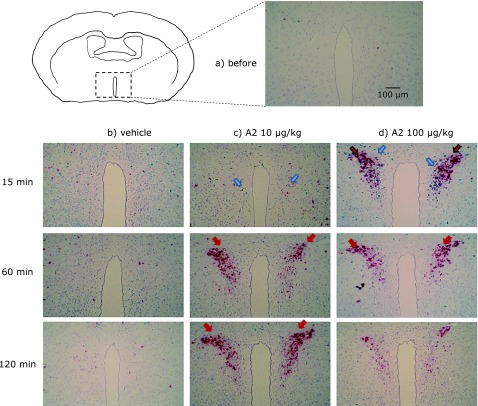
mRNA expression of c-fos and corticotropin-releasing hormone (CRH) detected by *in situ* hybridization inmouse paraventricular nucleus (PVN). Before treatment (a), after vehicle administration (b), after administration of 10 µg/kg (c) or 50 µg/kg A2 (d). Cells were stained with RNA probes to c-fos (blue) and CRH (red) mRNA sequences. Blue arrows indicate the adducts of c-fos; red arrows indicate the adducts of CRH mRNA.

**Fig. 3:**
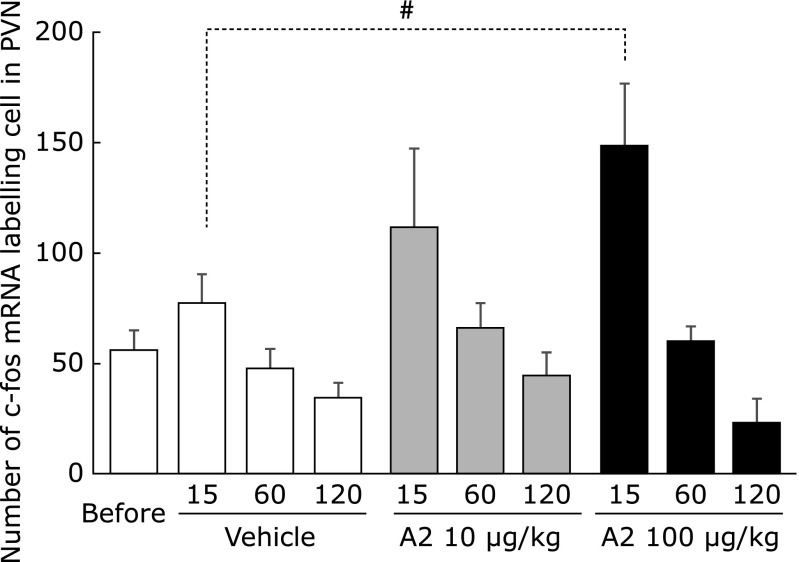
Quantitative results for c-fos mRNA levels in mouse paraventricular nucleus (PVN). Values represent the means ± SEM of each group (before; *n* = 8, vehicle; *n* = 6, 10 mg/kg A2 in 15 min; *n* = 4, in 60 min; *n* = 6, in 120 min; *n* = 4, 50 mg/kg A2 in 15 min; *n* = 6, in 60 and 120 min; *n* = 5). Significant differences from before treatment are indicated: ^#^*p*<0.1 vs vehicle group.

**Fig. 4:**
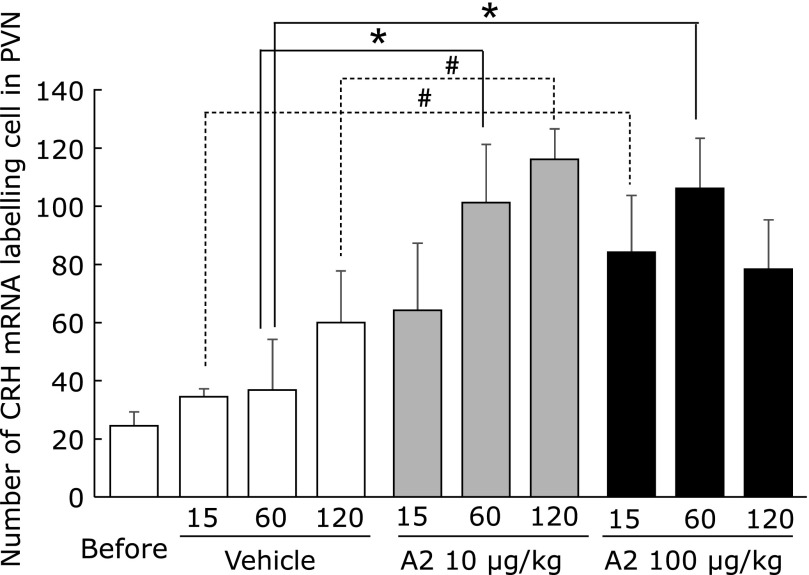
Quantitative results for corticotropin-releasing hormone (CRH) mRNA levels in mouse paraventricular nucleus (PVN). Values represent the means ± SEM of each group (before; *n* = 8, vehicle; *n* = 6, 10 mg/kg A2 in 15 min; *n* = 4, in 60 min; *n* = 6, in 120 min; *n* = 4, 50 mg/kg A2 in 15 min; *n* = 6, in 60 and 120 min; *n* = 5). Significant differences from before treatment are indicated: ^#^*p*<0.1, *p<0.05 vs vehicle group.

The *in situ* hybridization results (Fig. 2) showed the expression of c-fos mRNA was observed in the PVN at 15 min after a single dose of 10 or 100 µg/kg A2. At 60 or 120 min after a single dose of A2, the expression of c-fos mRNA was similar to that of the vehicle group. The CRH mRNA expression was present from 15 to 60 min after a single oral dose of 100 µg/kg A2, and then disappeared 120 min after administration. In the 10 µg/kg A2 group, the expression of CRH mRNA was observed at 60 and 120 min after the treatment period. The numbers of cells in the PVN that stained for c-fos are shown in Fig. 3. The expression of c-fos mRNA had a tendency to increase after 15 min in the 100 µg/kg A2 group compared with the vehicle group. The numbers of cells in the PVN that stained for CRH mRNA are shown in Fig. 4. The CRH mRNA expression levels tended to increase at 15 min and significantly increased, compared with the vehicle group, 60 min after a single oral administration of 100 µg/kg A2. The 10 µg/kg A2 group had significantly elevated CRH mRNA levels 60 min after the treatment period compared with the vehicle group.

## Discussion

CRH is known to play a key role in the integration of neuroendocrine, autonomic, and behavioral responses to stress.^(24)^ Neurons in the PVN release CRH into blood vessels that connect the hypothalamus to the pituitary gland, which produces and secretes adrenocorticotropic hormone into the general circulation.^(25)^ c-Fos is a proto-oncogene, which is expressed in some neurons following depolarization.^(26)^ Therefore, mRNA expression of c-fos is considered a marker of neuronal cell activation. Accordingly, c-fos staining is an extremely useful technique for detecting functional neuroendocrine systems.^(27)^

In the present study, c-fos mRNA was slightly increased 15 min after a single oral administration of 100 µg/kg A2 (Fig. 2 and 3). The CRH mRNA was significantly upregulated after administration of both 10 and 100 µg/kg A2 (Fig. 2 and 4) at 60 min. These results confirmed our hypothesis that A2 treatment activates neurons in the PVN and induces stress responses. When comparing the A2 treatment groups, CRH upregulation was observed earlier with 100 µg/kg A2 (from 15 to 60 min) than 10 µg/kg (from 60 to 120 min). In a previous study, after a single oral dose of flavan 3-ols, containing (−)-epicatechin and procyanidins, there was a rise in CRH mRNA expression in the PVN that was observed earlier with the 50 mg/kg dose than the 10 mg/kg dose.^(23)^ Stressor intensity is a major factor in determining the overall trajectory of the HPA axis response.^(28)^ However, the relationship between stress intensity and the following response remains unclear. Examination of two different intensities of restraint stress (0.5 h vs 3 h per day) revealed intensity-related increases in CRH mRNA expression in the PVN.^(29)^ Further studies are necessary to elucidate the relationship between stress intensity and the resulting response. Our results suggest that stress intensities induced by the different amounts of A2 regulated the duration until the stress response occurred.

It is well known that B type procyanidins, a series of (−)-epicatechin oligomers, are abundant in cocoa and dark chocolate.^(30,31)^ The A2 tetramer is a minor component among these procyanidins, and it is present in the range of 0.124–0.86 mg/g in dark chocolate.^(31,32)^ Considering the results of the present study, a stress response occurs in humans after ingesting 1–5 g of dark chocolate. Ingestion of procyanidins has shown significant potential in managing cardiovascular health, but the mechanism for these beneficial activities is still unknown. Our results clarify that A2 causes a stress response along with the activation of HPA and SAM axes. The activation of SAM axis induces beneficial adaptations for various chronic problems via sympathetic nerve, such as hypertension or obesity, through sympathetic hyperactivity.^(33)^ It seems that the stress response induced by procyanidins may contribute to reducing the risks for these chronic diseases.

It has been reported that mood, cognitive performance, and working memory were improved soon after a single dose of procyanidin-rich foods, along with increases in cerebral blood flow.^(9–12)^ In addition, procyanidins, including A2, had poor bioavailability.^(16–18)^ These findings suggest that procyanidins directly affect the central nervous system through characteristics, for instance its astringent taste, etc. Procyanidin-rich foods are also known to enhance flow mediated dilatation^(34–36)^ or insulin sensitivity^(37,38)^ a few hours after administration. It is rational that these alterations occurred as a result of procyanidin ingestion eliciting a stress response and promoting sympathetic nerve activity. In addition, A2 had potent metabolic activity compared with the other procyanidins.^(19–21)^

In conclusion, stress response induction mechanisms by A2 remains unknown. Further experiments are needed to clarify this, especially, it is needed to elucidate a target molecule in the gastrointestinal tract that recognizes A2, since A2 suggested to have a particular affinity for it. In conclusion, the result that A2 is recognized as a stressor of the central nervous system after digestion supported our hypothesis. The beneficial activities of A2 on circulation or metabolism reported previously were possibly the results of induced stress responses.

## Abbreviations

A2: cinnamtannin A2
CRH: corticotropin-releasing hormone
HPA: hypothalamic-pituitary-adrenal
PFA: paraformaldehyde
PVN: paraventricular nucleus
SAM: sympathetic-adrenal-medullary
